# Controlling Persister and Biofilm Cells of Gram-Negative Bacteria with a New 1,3,5-Triazine Derivative

**DOI:** 10.3390/ph8040696

**Published:** 2015-10-10

**Authors:** Ali Adem Bahar, Zhigang Liu, Meagan Garafalo, Neville Kallenbach, Dacheng Ren

**Affiliations:** 1 Department of Biomedical and Chemical Engineering, Syracuse University, Syracuse, NY 13244, USA; E-Mails: abahar@syr.edu (A.A.B.); meagan8390@yahoo.com (M.G.); 2 Syracuse Biomaterials Institute, Syracuse University, Syracuse, NY 13244, USA; 3 Department of Chemistry, New York University, New York, NY 10003, USA; E-Mail: zl264@nyu.edu; 4 Department of Civil and Environmental Engineering, Syracuse University, Syracuse, NY 13244, USA; 5 Department of Biology, Syracuse University, Syracuse, NY 13244, USA

**Keywords:** antimicrobial peptide, biofilm, persister cells, TN-5, *Escherichia coli*, *Pseudomonas aeruginosa*

## Abstract

Infections caused by multidrug-resistant bacteria have been on the rise. This important issue presents a great challenge to the healthcare system and creates an urgent need for alternative therapeutic agents. As a potential solution to this problem, antimicrobial peptides (AMPs) have attracted increasing attention due to their broad spectrum of targeted microbes. However, most AMPs are expensive to synthesize, have relatively high cytotoxicity to mammalian cells, and are susceptible to proteolytic degradation. In order to overcome these limitations, novel synthetic AMPs are desired. Using 1,3,5-triazine (TN) as a template, several combinatorial libraries with varying cationic charge and lipophilicity were designed and screened by the Kallenbach lab. From this screening, TN-5 was identified as a potent lead. In the present study, this compound was tested for its antimicrobial activities on *Escherichia coli* and *Pseudomonas aeruginosa*. In addition to regular planktonic cells, the effects on biofilms and persister cells (metabolically inactive and antibiotic tolerant subpopulation) were also investigated. TN-5 was found to have a minimum inhibitory concentration (MIC) of 12.8 µM for both species and kill regular planktonic cells of both species dose dependently. TN-5 is also effective against persister cells of both *E. coli* and *P. aeruginosa*. The killing of biofilm cells of the mucoid *P. aeruginosa* PDO300 was enhanced by alginate lyase.

## 1. Introduction

Since the discovery of penicillin in 1928 [1] and the achievement of economical production of this antibiotic in the 1940s [2], the use of antibiotics has been a crucial step in controlling infectious diseases with numerous lives saved [3]. However, the emergence and spread of antibiotic resistant microorganisms have rendered many antibiotics ineffective [4]. Such rapid development of multidrug resistant bacteria coupled with the insufficient investment in antimicrobial research has led to a concerning decline in effective therapies against bacterial infections, which presents a serious public health problem [5].

In addition to antibiotic resistance based on drug resistance genes [6], bacteria also exhibit high level antibiotic tolerance by forming persister cells (dormant subpopulation of phenotypic variants [7] and biofilms (surface attached structures with bacterial cells embedded in an extracellular matrix secreted by attached cells [8]). Persister cells and biofilms are not based on drug resistance genes; however, they allow bacteria to survive the treatment with potent antibiotics and facilitate the development of drug resistant strains through mutation and horizontal gene transfer [9].

According to the U.S. Centers for Diseases Control and Prevention, 65% of all infections in developed countries are caused by biofilms [10]. The biofilm matrix serves as a protective barrier, making bacterial cells more tolerant to antibiotics as well as host defense [11]. Biofilms are also enriched in persister cells. Thus, even if an antibiotic can penetrate the biofilm matrix, it might only kill normal cells within a biofilm population. After the course of antibiotic treatment, persister cells revive and repopulate the biofilm, which in turn causes an infection to relapse [10].

An additional concern with biofilms of *Pseudomonas aeruginosa* is their ability to convert to mucoid variants [12]. A mucoid strain is characterized by overproduction of the exopolysaccharide alginate, with increased tolerance to some antibiotics [13] and phagocytosis by human macrophages [14], as well as enhanced protection from dehydration [15]. In cystic fibrosis patients, the leading cause of mortality is a respiratory failure due to chronic lung infection with *P. aeruginosa* strains that undergo mucoid conversion [12]. Mucoid isolates typically coincide with persistent chronic infection in cystic fibrosis patients.

The challenges of drug tolerant infections have created an urgent need for new antimicrobials and treatment strategies. Recent research has shown the great potential of antimicrobial peptides (AMPs) as a class of powerful agents against both Gram-positive and Gram-negative bacteria [16,17]. A wide range of AMPs are naturally produced by the innate immune system of multicellular organisms in response to infections [18]. These AMPs are a unique group of molecules with a varying number of amino acids (generally from 12 to 50), including positively charged residues (such as arginine, lysine, or histidine) and a large proportion of hydrophobic residues. In humans, AMPs are found mainly in the tissues and organs that are exposed to airborne pathogens [19].

The structure and charge of an AMP play a major role in the mechanism of its actions. AMPs are generally cationic molecules with both hydrophobic and hydrophilic faces [20]. This amphipathic characteristic helps these molecules to integrate into the lipid bilayer membranes [21]. Membrane integrity disruption (via interaction with negatively charged cell membrane), inhibition of macromolecule (protein, DNA and RNA) synthesis, or interaction with certain intracellular targets are thought to be the primary mechanisms in AMP lethality [22]. Positively charged side chains in AMPs enable an initial nonspecific electrostatic interaction with the negatively charged cell membrane. This process is followed by the insertion of AMP molecule into cell membrane with the help of hydrophobic residues [23]. For example, in the AMPs LL-37 and β-defensin, the cationic face is positioned on the opposite side of the hydrophobic face, which helps the penetration into the membrane [20].

AMPs afford promising candidates for novel therapeutic agents and complement traditional antibiotic therapies because of some unique advantages, including broad-spectrum activity (antibacterial, antiviral, and antifungal), less resistance by microbes, and related broad anti-inflammatory activities [24]. A number of AMPs and derivatives have been developed as therapies for infectious diseases such as oral mucositis [25], pulmonary infections associated with cystic fibrosis [26], and some sexually transmitted diseases [27]. AMPs can be used alone, in co-treatment with antibiotics, or as stimulators for immune system and toxin inhibiting agents in septic shocks [24].

Despite the aforementioned advantages, wide applications of AMPs are still limited by several factors. They are generally expensive to synthesize [28], vulnerable to proteolytic degradation upon intravenous administration [29] and sensitive to environmental factors such as salt concentration, pH, and the presence of plasma and serum proteins [30]. Another challenge is that some AMPs are cytotoxic to host cells [31].

**Figure 1:**
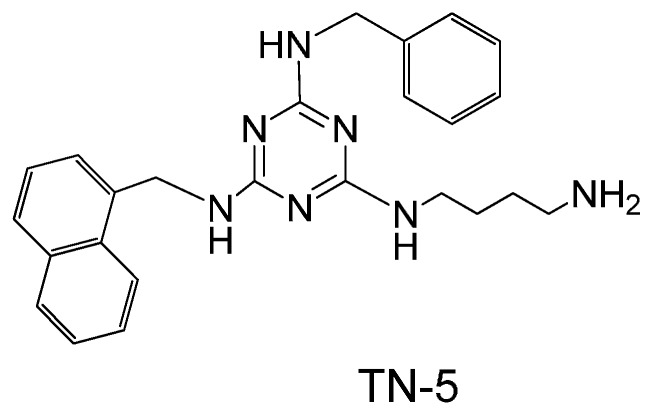
Chemical structure of TN-5.

In order to overcome these challenges, a number of synthetic AMPs and AMP-mimetics have been developed. Based on the concept that cationic charge, size, and lipophilicity are recognized major factors determining the antibacterial activity of AMPs, recently, the Kallenbach lab designed and screened several combinatorial libraries based on 1,3,5-triazine as a scaffold. Several lead compounds with good antimicrobial activity and low hemolytic activity were identified from the screening of hundreds of triazine compounds [32]. With further structure activity relationship analysis, the compound TN-5 was identified as a potent antimicrobial compound (the screening results will be published elsewhere). In this study, TN-5 (Figure 1) was tested for its antimicrobial activity on *E. coli* and *P. aeruginosa*, including regular planktonic cells, persister cells, and biofilms.

## 2. Results

### 2.1. Minimum Inhibitory Concentration (MIC) and Minimal Bactericidal Concentration (MBC) of TN-5

TN-5 was found to completely inhibit the growth of *E. coli* RP437, *P. aeruginosa* PAO1, and *P. aeruginosa* PDO300 at the concentration of 12.8 µM (MIC, Table 1). The MBC value was found to be higher than 96 µM for all three strains.

**Table 1:** Minimum Inhibitory Concentration (MIC) and Minimal Bactericidal Concentration (MBC) values of TN-5 on bacterial strains used in this study (based on three biological replicates).

Antimicrobial Test	Bacterial Strains		
	*E. coli* RP437	*P. aeruginosa* PAO1	*P. aeruginosa* PDO300
MIC (µM)	12.8	12.8	12.8
MBC (µM)	>96	>96	>96

### 2.2. Antimicrobial Effects of TN-5 on Planktonic Cells

**Figure 2:**
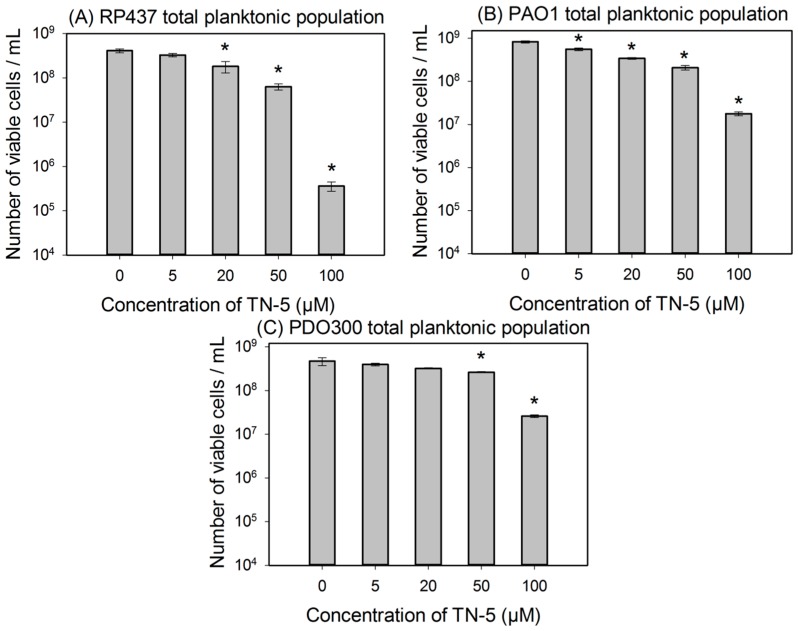
Effects of TN-5 on planktonic cells of *E. coli* RP437 (**A**), *P. aeruginosa* PAO1 (**B**), and *P. aeruginosa* PDO300 (**C**). TN-5 was added in exponential phase cultures at different concentrations and the viability after treatment was determined by counting colony forming unit (CFU). All significant differences (compared to the TN-5 free control) with *p* < 0.01 are marked with an asterisk.

To further study the killing activity of TN-5, exponential cultures were used to test the effects of TN-5 on the viability of planktonic cells. TN-5 was found effective in killing all bacterial strains tested in this study (*E. coli* RP437, *P. aeruginosa* PAO1 and PDO300) dose dependently in 3 h (Figure 2). Longer incubation times (up to 24 h) did not cause additional killing (Figure S1). For *E. coli* RP437, TN-5 showed 20.3% ± 2.5% (*p* = 0.07), 55.6% ± 8% (*p <* 0.001), 84.6% ± 8.5% (*p <* 0.001), and 99.9% ± 0.1% (3.4 log; *p <* 0.001) killing of the total population (>99% as normal cells) at concentrations of 5, 20, 50, and 100 µM, respectively (Figure 2A). These results show that TN-5 is highly effective against normal planktonic cells of *E. coli* RP437.

Significant killing effects were also observed on planktonic cells of *P. aeruginosa* PAO1 and PDO300 strains. For example, killing of 22.7% ± 6.7% (*p <* 0.001), 61.8% ± 4.5% (*p <* 0.001), 74.7% ± 11.8% (*p <* 0.001), and 97.8% ± 10% (1.6 log; *p <* 0.001) of *P. aeruginosa* PAO1 was observed when TN-5 was added at 5, 20, 50, and 100 µM respectively (Figure 2B). For *P. aeruginosa* PDO300, significant killing was observed at 50 and 100 µM with 44.1% ± 3.1% (*p <* 0.001) and 94.1 ± 6.8% (1.23 log; *p <* 0.001) of the total population (>99% as normal cells) killed, respectively (Figure 2C). These results show that TN-5 is also effective against normal planktonic cells of *P. aeruginosa*.

### 2.3. Antimicrobial Effects of TN-5 on Persister Cells

**Figure 3:**
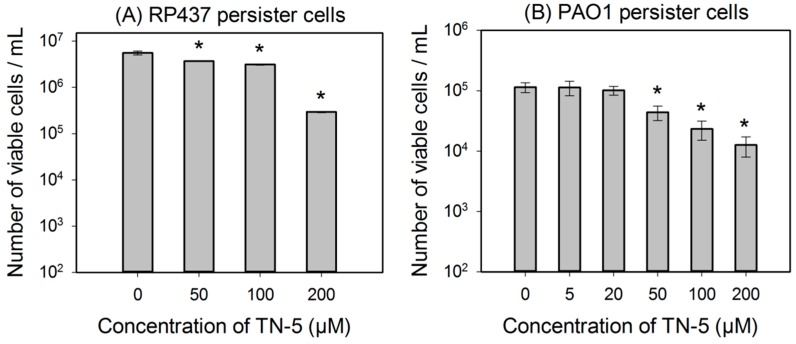

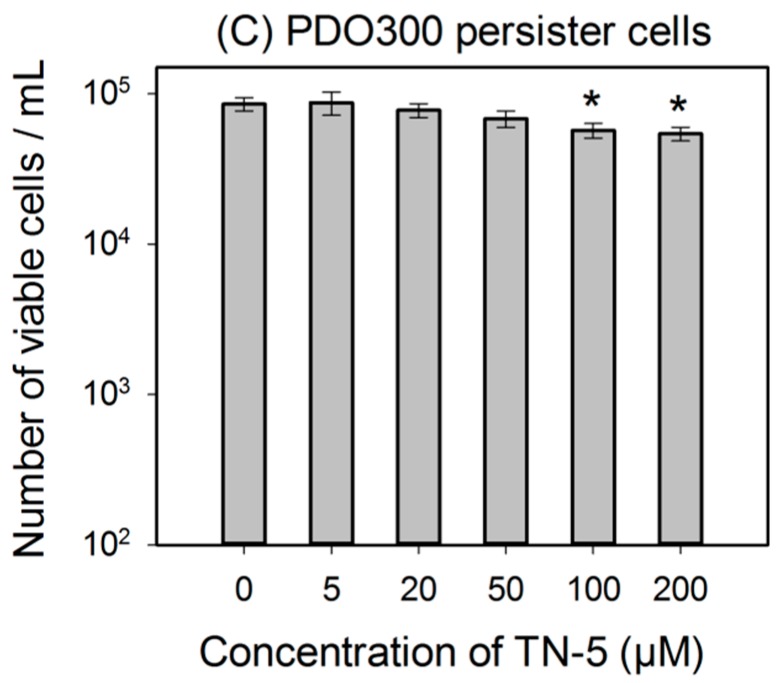
Effects of TN-5 on persister cells of *E. coli* RP437 (**A**), *P. aeruginosa* PAO1 (**B**), and PDO300 (**C**). The persister cells were isolated by treating exponential cultures of *E. coli* RP437 with 100 µg/mL ampicillin and overnight *P. aeruginosa* cultures with 200 µg/mL ciprofloxacin (both for 3 h). All significant differences (compared to the TN-5 free control) with *p* < 0.01 are marked with an asterisk.

TN-5 was found to kill persister cells of *E. coli* RP437, *P. aeruginosa* PAO1 and *P. aeruginosa* PDO300 (Figure 3). The killing of *E. coli* RP437 persister cells was 33.8% ± 0.8% (*p <*0.001), 43.8% ± 2.8% (*p <* 0.001), and 96.3% ± 3.0% (1.35 log; *p <* 0.001) when TN-5 was added at 50, 100, and 200 µM, respectively (Figure 3A).

Similarly, TN-5 was also able to reduce the viability of *P. aeruginosa* PAO1 persister cells where 79.6% ± 2.9% (*p* < 0.001) and 89.9% ± 4.5% (*p* < 0.001) of cells were killed by 100 μM and 200 μM of TN-5, respectively (Figure 3B). In comparison, TN-5 was less effective on persister cells of the mucoid strain *P. aeruginosa* PDO300, with only 33.2% ± 5.6% (*p* < 0.001) and 36.4% ± 5.2% (*p* < 0.001) killed by 100 μM and 200 μM of TN-5, respectively (Figure 3C). This is likely due to the presence of alginate on the surface of the mucoid *P. aeruginosa* PDO300 cells [33], which may reduce the penetration by TN-5.

### 2.4. Antimicrobial Effects of TN-5 on Biofilm Cells

**Figure 4:**
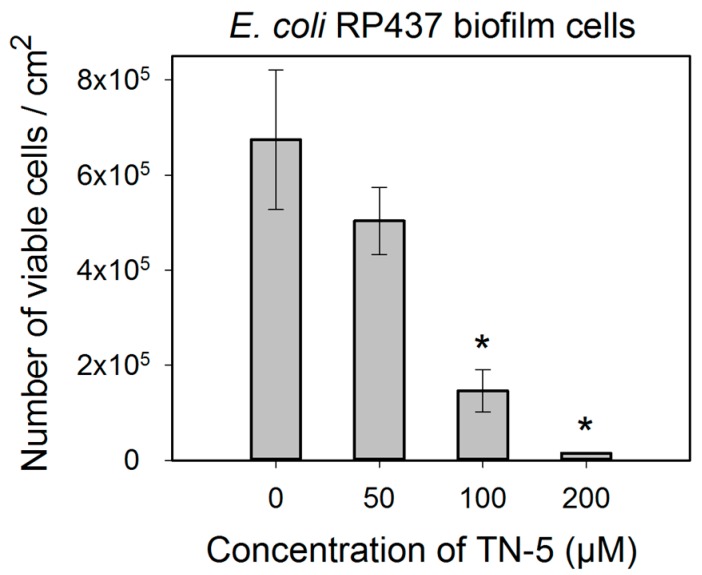
Effects of TN-5 on *E. coli* RP437 biofilms. The biofilms were cultured for 24 h in LB on stainless steel coupons prior to treatment with TN-5. All significant differences (compared to the TN-5 free control) with *p* < 0.01 are marked with an asterisk. Note: Data are plotted in linear scale.

**Figure 5:**
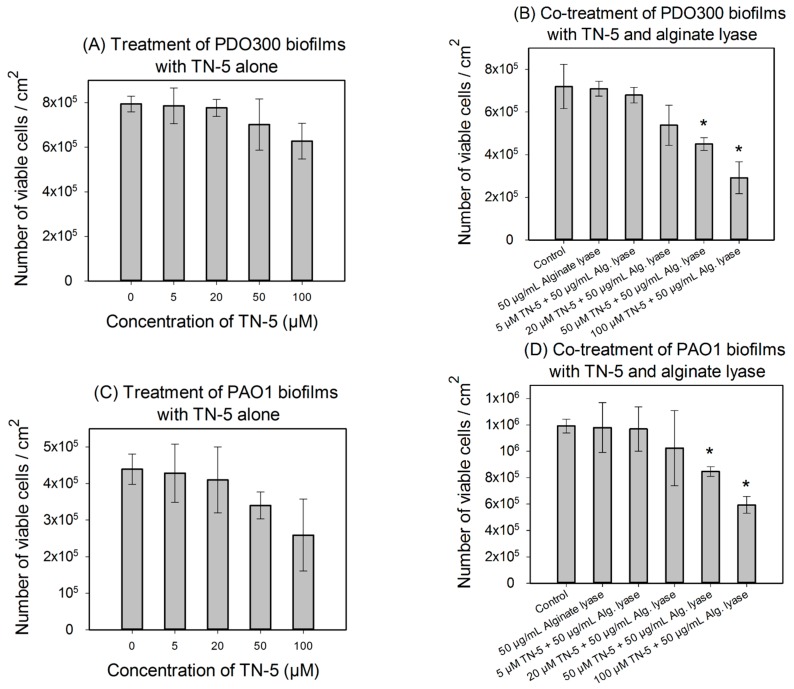
Effects of TN-5 alone and co-treatment with alginate lyase on *P. aeruginosa* PDO300 (**A** and **B**) and PAO1 (**C** and **D**) biofilms. The biofilms were grown for 24 h and treated with TN-5 alone or in combination with alginate lyase for 3.5 h. All significant differences (compared with the TN-5 free control) with *p* < 0.01 are marked with an asterisk. The co-treatment with TN-5 (at 50 or 100 µM) and 50 µg/mL alginate lyase caused significant killing compared to the control (with no TN-5 and alginate lyase) for both *P. aeruginosa* PDO300 and PAO1, but only significantly increased the killing by TN-5 alone for *P. aeruginosa* PDO300. Note: Data are plotted in linear scale.

TN-5 exhibited effective and dose dependent killing of *E. coli* RP437 biofilms. Killing of 78.5% ± 30.2% (*p <* 0.001) and 98.9% ± 9.6% (1.24 log; *p <* 0.001) of *E. coli* RP437 biofilm cells was achieved when TN-5 was added at 100 and 200 µM, respectively (insignificant at 50 µM) (Figure 4). In addition to the effects on established biofilms of *E. coli* RP437, TN-5 effectively prevented biofilm formation of all the strains used in this study. Complete biofilm inhibition of *E. coli* RP437 and *P. aeruginosa* (both PAO1 and PDO300) was achieved with 10 and 20 µM TN-5, respectively (Figure S2). However, 100 µM TN-5 alone did not show significant killing of cells in established biofilms of *P. aeruginosa* PAO1 or PDO300 (*p* > 0.1) (Figure 5A,C).

We speculated that the lack of antimicrobial effects against *P. aeruginosa* biofilms is because of the presence of biofilm matrix. Alginate is a major component of biofilm matrix of the mucoid *P. aeruginosa* strains [13,33]. Thus we tested the concurrent treatment of *P. aeruginosa* biofilms with alginate lyase and TN-5. As shown in Figure 5B,D, alginate lyase itself showed no effect on the viability of biofilm cells (*p* = 1). However, the combination of TN-5 with alginate lyase increased the killing of PDO300 biofilms; e.g., addition of 50 µM alginate lyase increased the activity of 50 µM TN-5 on *P. aeruginosa* PDO300 biofilms from no significant killing (*p* = 0.58) to 36.5% ± 4.6% (*p <* 0.005) and that of 100 µM TN-5 from insignificant killing (*p* = 0.12) to 57% ± 9.6% (*p <* 0.005), respectively (Figure 5A *vs*. Figure 5B). The addition of alginate lyase did not show the same effects on *P. aeruginosa* PAO1 biofilm cells; e.g., 50 µM alginate lyase did not increase the effects of 50 and 100 µM TN-5 (*p* > 0.1) (by comparing the % of killing based on corresponding data between Figure 5C,D). This is likely because alginate is not a major component of its biofilm matrix.

## 3. Discussion

AMPs have been proposed as a promising source of new antimicrobial agents [34,35,36,37]. Different strategies have been tested to achieve effective killing of microbes while maintaining low hemolytic activity. In some of these studies, Trp and Arg containing 1,3,5-triazine structures [32] and dendrimeric peptides [38] have been used as AMP templates and lead compounds with antimicrobial activities and low toxicity to red blood cells have been identified. In this study, one of the triazine-1,3,5 derivatives, TN-5, was tested on Gram-negative bacteria *E. coli* and *P. aeruginosa*. TN-5 was found to be effective against both species. The killing of persister and biofilm cells is of particular interest because these cells are difficult to eliminate and conventional antibiotics are generally ineffective. Our results show that TN-5 alone is effective against persister and biofilms of *E. coli* RP437 with up to 2-log killing achieved at 200 µM. It is also interesting that TN-5 caused more killing of biofilm cells than planktonic persister cells of *E. coli*. For example, 100 µM TN-5 killed 43.8% ± 2.8% and 78.5% ± 30.2% of persister and biofilm cells of *E. coli* RP437, respectively. This finding suggests that TN-5 can penetrate the biofilm matrix of *E. coli*.

Comparable effects were not observed for *P. aeruginosa* biofilms, however, possibly due to the difference in biofilm matrices. With a thick layer of alginate, biofilms of mucoid bacteria have high-level tolerance to some antimicrobials [13]. Thus, breaking down alginate in the biofilm matrix could be essential for certain antimicrobials to kill biofilm cells, especially the agents that can be absorbed or neutralized by the matrix components. Consistently, we found that the killing of *P. aeruginosa* PDO300 biofilm cells was enhanced by alginate lyase. It is worth noting that TN-5 is effective against *P. aeruginosa* PDO300 persister cells, but not its biofilm cells. Thus, it will be interesting to study if and how TN-5 interacts with alginate directly and if there is any difference in the amount and structure of alginate between biofilm matrix and the surface of mucoid cells. In comparison, the killing of *P. aeruginosa* PAO1 biofilms was not enhanced by alginate lyase. This is consistent with the report [39] that alginate is not the primary component of *P. aeruginosa* PAO1 biofilm matrix. It will be interesting to test other matrix degrading enzymes, such as DNase.

In a previous study performed in our labs, Trp- and Arg- containing synthetic AMPs were shown to inhibit 95.0% ± 1.1% of *E. coli* biofilm formation at 200 µM [40]. Intriguingly, TN-5 inhibited biofilm formation of both *E. coli* and *P. aeruginosa* completely at 20 µM (Figure S2). Therefore, TN-5 may also be an important antimicrobial agent for biofilm control.

The mechanism of bacterial killing by TN-5 or in fact any AMP deserves more study. The positive charge of the arginine (R) side chain can help an AMP to interact with negatively charged lipopolysaccharides in Gram-negative bacterial walls [41]. Tryptophan (W) residues favor location below the head groups of bilayers, and are assumed to provide lipophilic interaction sites to cause membrane disruption [42]. Thus, RW mimicking TN-5 might target the negatively charged bacterial cell membrane. Another observation is that TN-5 mode of action occurs within the first 3 h (Figure S1). This rapid mode of action is one of the major features of membrane acting AMPs.

*E. coli* is generally more susceptible to antibiotics than *P. aeruginosa*. Among 47 antimicrobial agents tested in an early study [43], only tobramycin showed the same MIC value (0.5 µg/mL) for *E. coli* (NCTC 10418 and ATCC 25922) and *P. aeruginosa* (NCTC 10662 and ATCC 27853) reference strains. The other antimicrobial agents tested all showed higher (up to 500 fold) MIC values for *P. aeruginosa* compared to *E. coli* [43]. Interestingly, TN-5 showed the same MIC value (12.8 µM) for both species in our study, which also indicates possible membrane targeting activities.

Overall, this study shows that the triazine derivative TN-5 is a promising lead compound for developing new synthetic AMPs. It is encouraging that TN-5 is effective against both *E. coli* and *P. aeruginosa* at different growth stages. Persister cells of *Pseudomonas* strains, especially those of the mucoid strains, are highly resistant to antibiotic treatments. Because most AMPs kill microbes through mechanisms that differ from those of conventional antibiotics, e.g. by targeting cell membranes, the killing effect of TN-5 on persister cells might be increased by synergy with some antibiotics if TN-5 can be effectively delivered to target cells. We assume that triazines are not susceptible to proteolytic degradation since there is no specific motif to trigger cleavage. Future experiments with mammalian cells and animal models are needed to evaluate the potential of using TN-5 and antibiotics together as a novel therapy for chronic infections involving biofilms and persister cells.

## 4. Experimental Section

### 4.1. Chemical Synthesis of TN-5

TN-5, *N*2-(4-Aminobutyl)-*N*4-benzyl-*N*6-naphthalenemethyl-2,4,6-triamino-1,3,5-triazine (Figure 1), white solid, was synthesized using an orthogonal synthetic approach based on cyanuric chloride stepwise reaction with naphthalenemethylamine, benzylamine and Boc-1,4-diaminobutane as documented previously [32]. In brief, (i) cyanuric chloride was mixed with naphthalenemethylamine (1.1 eq) in tetrahydrofuran (THF) at 0 °C, and N,N-diisopropylethylamine (DIEA) (2 eq). After 1 hour of stirring, the solution was extracted with water/ethyl acetate and product 2, 4-dichloro-*N*6-naphthalenemethylamino-1,3,5-triazine was purified using flash column chromatography. (ii) PAL aldehyde resin was mixed with benzylamine (5 eq.) in THF with 2% of acetic acid and shaked at room temperature for 1 h. At the end of the hour, NaB (OAc) 3H (7 eq.) was added and mixed at room temperature overnight. (iii) The product on resin (4 eq) was mixed with 2, 4-dichloro-*N*6-naphthalenemethylamino-1,3,5-triazine in THF and heated at 60 °C for 3 h. (iv) Resin from last step was mixed with Boc-1,4-Diaminobutane (10 eq) and DIEA (30 eq) in *N*-methylpyrrolidinone-*N*-butanol (NMP:*n*-BuOH) (1:1) and heated for 3 h at 120 °C. (v) Resin was cleaved with 10% TFA in dichloromethane (DCM) for 30 min. Final product was dried and purified with prep thin layer chromatography (TLC). The identity of TN-5 was confirmed by LC-MS (Agilent 6226 TOF LC/MS Mass Spectrometer, Agilent Technologies Inc., Santa Clara, CA, USA). HRMS *m*/*z*: calculated [M + H]^+^ 428.2557, found 428.2566 (Figure S3). The composition of the product was verified by ^1^H-NMR (300 MHz, CD_3_OD): δ 8.10–7.86 (m, 1H), δ 7.8–7.65 (m, 2H), δ 7.55–7.30 (m, 4H), δ 7.30–7.15 (m, 3H), δ 7.18 (s, 2H), δ 5.05–4.92 (m, 2H), δ 4.55–4.40 (m, 2H), δ 3.40–3.25 (m, 2H), δ 2.90-2.61 (m, 2H), δ 1.65–1.40 (m, 4H) (Figure S4).

### 4.2. Bacterial Strains and Growth Media

*E. coli* RP437 [44] was provided by Dr. John S. Parkinson at the University of Utah. *P. aeruginosa* PAO1 [45] and *P. aeruginosa* PDO300 [12] were obtained from Dr. Matthew Parsek at the University of Washington. All strains were routinely grown in Lysogeny broth (LB) containing 10 g/L NaCl, 5 g/L yeast extract, and 10 g/L tryptone with pH 7.0. To ensure consistent experimental conditions throughout this study, all overnight cultures of a particular strain were started with single-use glycerol stocks originating from the same culture. Each experimental condition was tested with three independent cultures (three biological replicates).

### 4.3. MIC and MBC Values of TN-5

TN-5 was tested at different concentrations against *E. coli* and *P. aeruginosa* strains to determine MIC and MBC values following a previously described protocol [46] with slight modifications. MIC is referred to the concentration of an antimicrobial agent, which inhibits the visible growth of a given bacterium completely by checking with unaided eye. MBC is referred to the minimum concentration of an antimicrobial agent required to completely kill all the tested bacterial cells of a particular strain by checking colony formation on the plates after treatment. Briefly, exponential cultures of bacterial samples were used to inoculate test samples with a cell density of 5 × 10^5^ colony forming unit (CFU)/mL. TN-5 was tested at concentrations from 0.2 µM to 96 µM, increasing logarithmically. Cultures grown without antimicrobial and sterile LB medium were used as positive and negative controls, respectively. All samples were adjusted to a final volume of 3 mL and incubated for 16–18 h at 37 °C. After incubation, the concentrations with no visible growth were assigned by visual check to determine MIC. MBC value of TN-5 was identified by spreading TN-5 (0.2 µM to 96 µM) treated cells (overnight in LB) on LB agar plates and checking for growth after 24 h. Three independent cultures for each concentration were tested for MIC and MBC tests.

### 4.4. Effects on Planktonic Cells

To examine the antimicrobial activity of TN-5, the planktonic growth of *E. coli* RP437, *P. aeruginosa* PAO1 and PDO300 with TN-5 added at different concentrations was examined. Overnight cultures of each strain were grown in 50 mL LB medium for 12–16 h at 37 °C. Subcultures were then prepared by inoculating LB medium with overnight cultures to an optical density at 600 nm (OD_600_) of 0.01 and harvested when the OD_600_ reached 0.4–0.5. These exponential cultures were washed with phosphate buffered saline (PBS) three times. Cell pellets were washed with fresh PBS and re-suspended in 20 mL PBS buffer. For the TN-5 treatment, 3 mL of each sample was taken and mixed with TN-5 at different concentrations. The samples were incubated for 3, 6, 12 and 24 h at 37 °C with shaking at 200 rpm, and washed three times with PBS. Then a serial dilution of each sample was performed and the cells were spread on LB agar plates for counting CFU. The amount of DMSO (solvent to dissolve TN-5 in stock solutions) was adjusted to be the same for all samples to eliminate any solvent effect. Each condition was tested with three independent cultures.

### 4.5. Persister Isolation and Treatment

To isolate persister cells, overnight cultures of *P. aeruginosa* PAO1 and *P. aeruginosa* PDO300 strains were treated with 200 µg/mL ciprofloxacin while exponential cultures of *E. coli* RP437 were treated with 100 µg/mL ampicillin (all for 3 h at 37 °C) to kill normal cells as described previously [47,48,49]. Then the persister cells were harvested by centrifugation, washed with PBS three times to remove remaining antibiotic, and re-suspended in 20 mL PBS. Aliquots (1 mL) of each sample were supplemented with TN-5 at different concentrations. Three replicates were tested for each condition and all samples were incubated for 3 h at 37 °C with shaking at 200 rpm. No change in persister cell number was found before and after incubation for 3 and 6 h in PBS without TN-5 (regardless the presence of antibiotics; Figure S5). After TN-5 treatment, the samples were washed with PBS three times and plated to count CFU as described above. Each condition was tested with three independent cultures.

### 4.6. Biofilm Experiments

To study the effects of TN-5 on biofilm cells of *E. coli* and *P. aeruginosa*, biofilms were grown on 2 cm × 1 cm 316L stainless steel coupons. Each coupon was polished with 220 Grit sandpaper (3M, Sandblaster, St. Paul, MN, USA) on both sides and sterilized with 70% ethanol for at least 15 min. The coupons were then dried in a 50 °C oven for 15 min. Sterilization was confirmed by incubating three coupons in LB medium for 24 h at 37 °C and checking the turbidity of the cultures. To culture biofilms, sterilized coupons were transferred into new sterile plates containing 20 mL LB medium. To initiate biofilm formation, each sample was inoculated to OD_600_ of 0.01 with an overnight culture. The coupons were incubated for 24 h at 37 °C without shaking to grow biofilms. After 24 h incubation the coupons were washed gently with PBS three times to remove planktonic cells. Coupons were then placed in 12 well plates separately, each including a different concentration of TN-5 in 2 mL PBS buffer. The coupons were incubated for 3 h at 37 °C with no shaking followed by washing with PBS three times. Each coupon was then transferred into a 15 mL sterile conical test tube containing 3 mL PBS. Samples were gently sonicated for 4 min in a water sonication bath (Branson B200 Ultrasonic, Danbury, CT, USA) and then vortexed for 15 s. Three replicates were tested for each condition. The cells in the suspension were then spread on LB agar plates to count CFU as described above. Each condition was tested with three independent cultures.

Besides treating established biofilms, the capability of TN-5 to prevent biofilm formation was also evaluated by adding TN-5 prior to the inoculation of biofilm cultures. After incubation for 24 h, the coupons were washed gently and the cells were removed from coupon surface by sonication and vortex for CFU count as described above.

### 4.7. Alginate Lyase Enhanced the Effects of TN-5 on P. aeruginosa Biofilm Cells

To determine the contribution of a biofilm degrading agent in bacterial killing by TN-5, we repeated biofilm killing experiments in the presence of 50 µg/mL alginate lyase during TN-5 treatments. The same protocols were used for *P. aeruginosa* PAO1 and PDO300 biofilms. Each condition was tested with three independent cultures.

### 4.8 Statistical Analysis

The data from CFU experiments were analyzed with one-way ANOVA followed by Tukey test using SAS version 9.4 (SAS Institute, Cary, NC, USA). Differences with *p* < 0.01 were considered as statistically significant.

## Supplementary Files

Supplementary File 1
