# Supplementary material for: Controlling Persister and Biofilm Cells of Gram-Negative Bacteria with a New 1,3,5-Triazine Derivative
Source: Pharmaceuticals (Basel). 2015 Oct 10;8(4):696–710. doi: 10.3390/ph8040696 (PMC4695806; doi:10.3390/ph8040696)
Supplement: Supplementary File 1 [file pharmaceuticals-08-00696-s001.pdf]

Supplementary

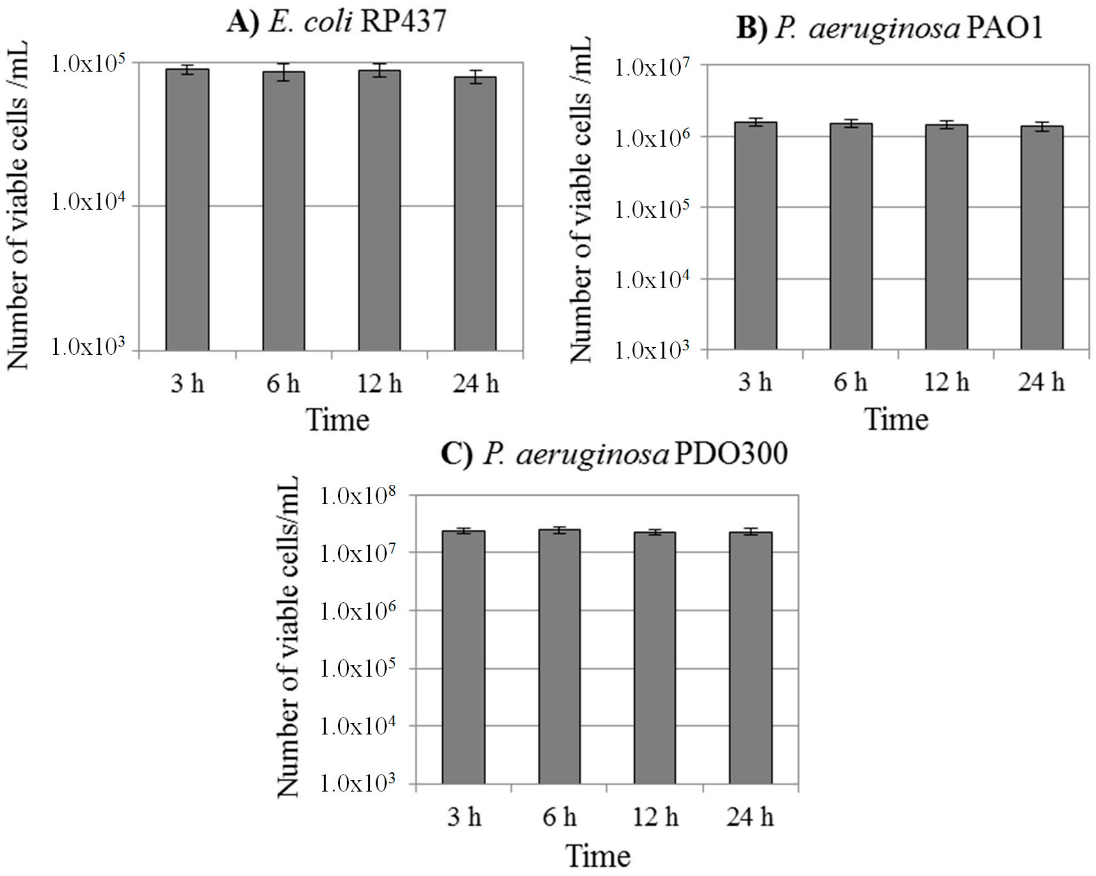

Figure S1. Extended treatment time did not increase the killing of planktonic cells of *E. coli* and *P. aeruginosa* by TN-5. Each sample was treated with 100 μM TN-5 for 3, 6, 12, or 24 h. Three independent replicates were tested for each condition. *E. coli* RP437 (**A**), *P. aeruginosa* PAO1 (**B**), and *P. aeruginosa* PDO300 (**C**) were studied.

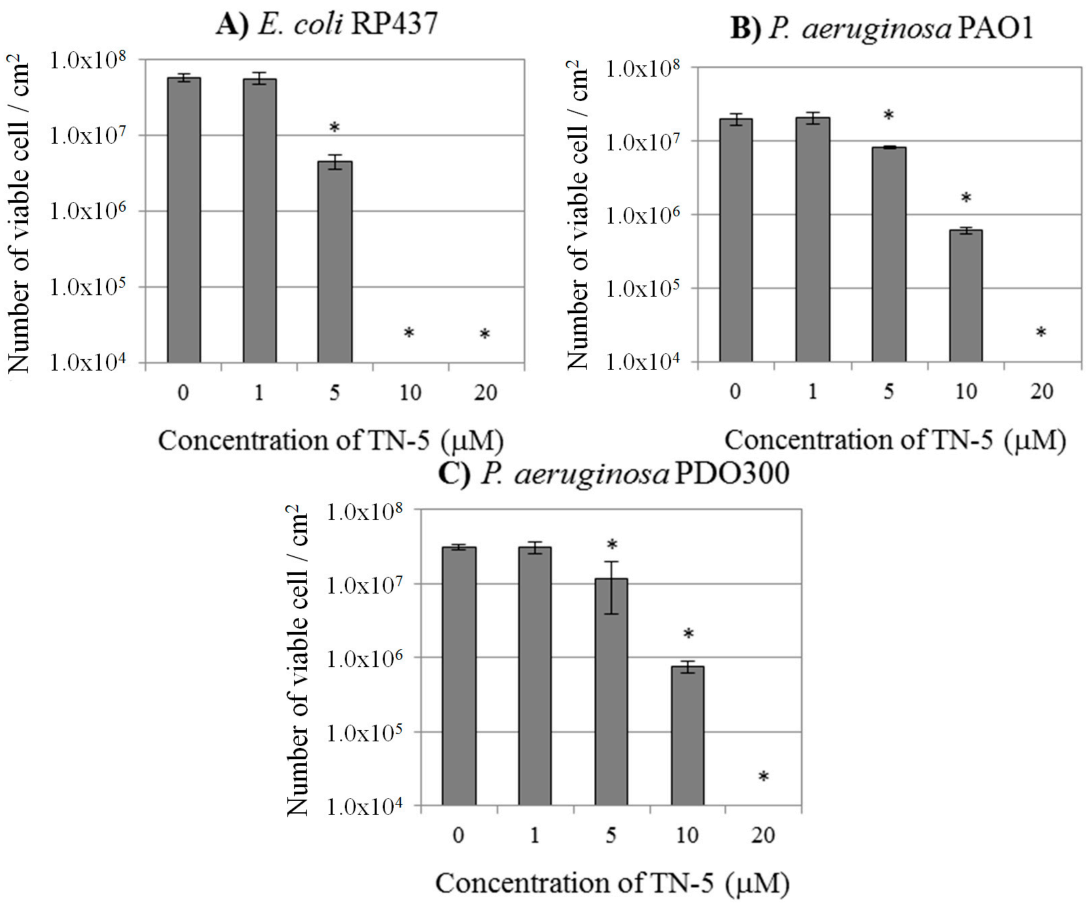

Figure S2. Effects of TN-5 on biofilm formation of *E. coli* RP437 (**A**), *P. aeruginosa* PAO1 (**B**), and *P. aeruginosa* PDO300 (**C**). Initial biofilm cultures were supplemented with TN-5 at different concentrations and incubated for 24 h at 37 °C. Then coupons were sonicated and vortexed to count CFU in biofilms. Three independent replicates were tested for each condition. All significant differences (compared with the TN-5 free control) with $p < 0.01$ are marked with an asterisk.

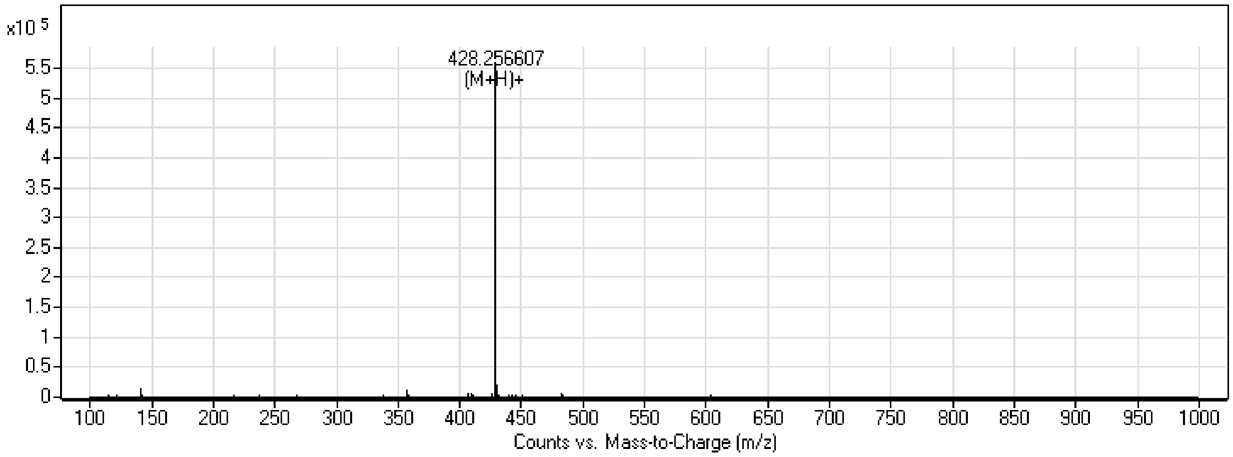

Figure S3. Mass spectrum of TN-5. The identity of TN-5 was confirmed by LC-MS (Agilent 6226 TOF LC/MS Mass Spectrometer). HRMS *m*/*z*: calculated $[M + H]^+$ 428.2557, found 428.2566, Δ = 2.1 ppm.

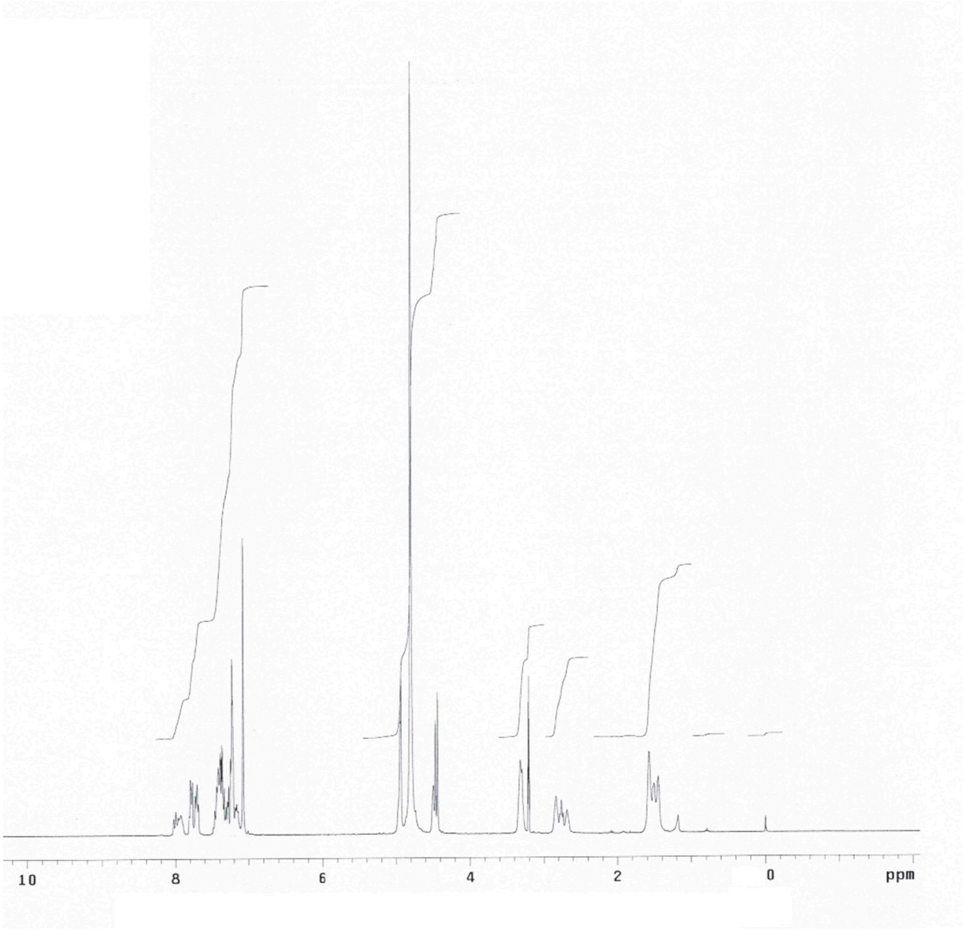

Figure S4. ^{1}H-NMR of TN-5. TN-5 was characterized by ^{1}H-NMR (Varian Mercury 300 MHz, CD3OD): δ 8.10–7.86 (m, 1H), δ 7.85–7.65 (m, 2H), δ 7.55–7.30 (m, 4H), δ 7.30–7.15 (m, 3H), δ 7.18 (s, 2H), δ 5.05–4.92 (m, 2H), δ 4.55–4.40 (m, 2H), δ 3.4–3.25 (m, 2H), δ 2.90–2.61 (m, 2H), δ 1.65–1.40 (m, 4H).

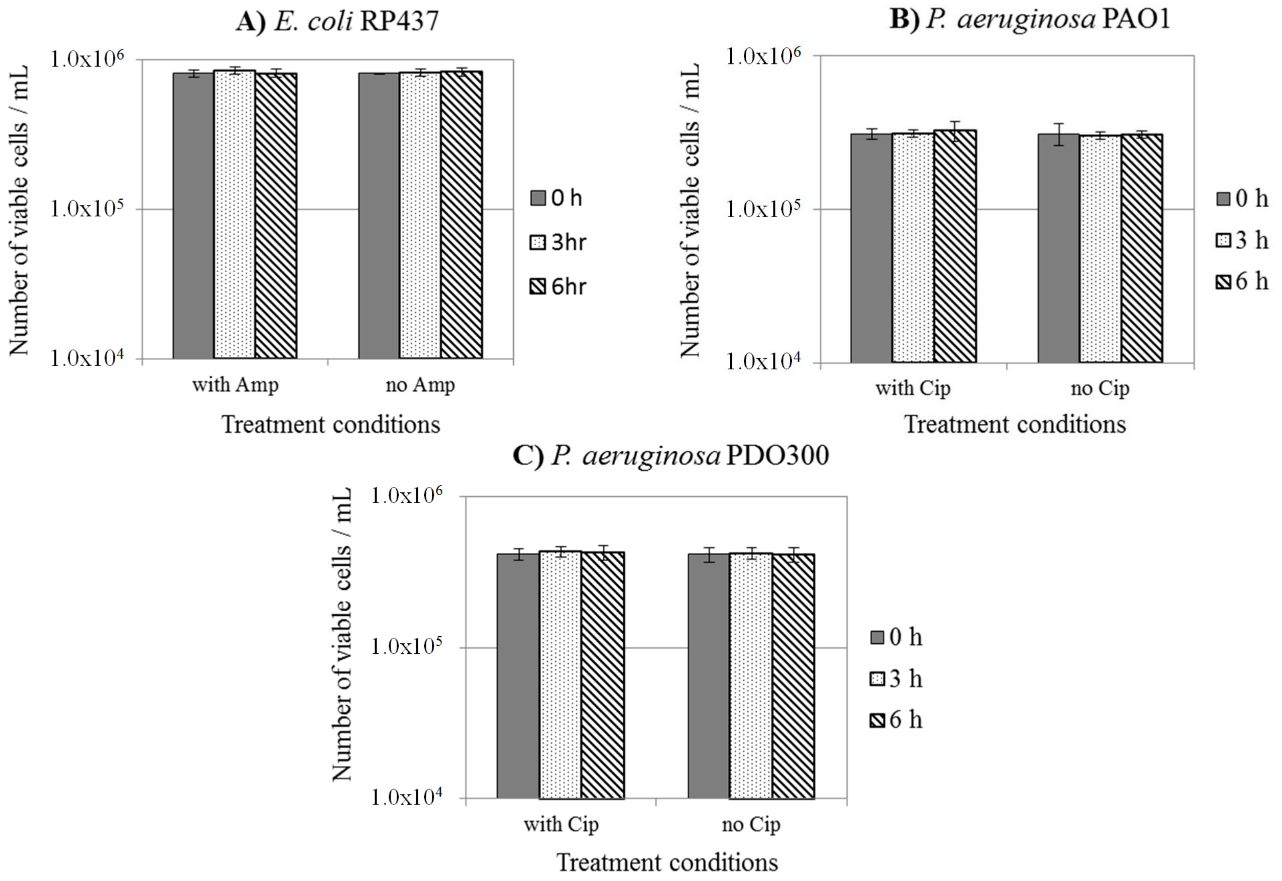

Figure S5. The isolated persister cells remained persistence after incubation in PBS. After isolation, the persister cells were incubated with or without antibiotic for 3 and 6 h. The persister cells of *E. coli* RP437 (**A**), *P. aeruginosa* PAO1 (**B**), and *P. aeruginosa* PDO300 (**C**) were studied. Ampicillin at 100 µg/mL and ciprofloxacin at 200 µg/mL were used to treat *E. coli* and *P. aeruginosa* persister cells, respectively.

© 2015 by the authors; licensee MDPI, Basel, Switzerland. This article is an open access article distributed under the terms and conditions of the Creative Commons Attribution license (http://creativecommons.org/licenses/by/4.0/).
